# Successful Vaginal Delivery in a Young Female With Eisenmenger Syndrome and Double Outlet Right Ventricle: A Challenging Case Report

**DOI:** 10.1155/cric/3877926

**Published:** 2026-07-26

**Authors:** Osman Farah Dahir, Said Abdirahman Ahmed, Ahmed Elmi Abdi, Ishak Ahmed Abdi, Ahmed Shafie Aden, Mohamed Mukhtar Mohamed, Mohamud Mire Waberi, Mohamed Omar Hassan

**Affiliations:** ^1^ Cardiology Department, Mogadishu Somali Türkiye Training and Research Hospital, Mogadishu, Somalia; ^2^ Faculty of Medicine and Surgery, Salaam University, Mogadishu, Somalia; ^3^ Emergency Department, Mogadishu Somali Türkiye Training and Research Hospital, Mogadishu, Somalia

**Keywords:** congenital heart defect, double outlet right ventricle, Eisenmenger syndrome, fetal outcomes, high-risk pregnancy, maternal outcomes, pulmonary hypertension

## Abstract

**Background:**

Eisenmenger syndrome (ES), a severe complication of uncorrected congenital heart defects, is characterized by pulmonary hypertension and right‐to‐left intracardiac shunting resulting in systemic hypoxemia. Double outlet right ventricle (DORV), a rare congenital anomaly where both great arteries arise predominantly from the right ventricle, further exacerbates hemodynamic instability. Pregnancy in women with ES and DORV is associated with extremely high maternal and fetal mortality rates, requiring meticulous, multidisciplinary management.

**Case Presentation:**

A 20‐year‐old Gravida 1 Para 0 woman at 34 weeks gestation presented with progressive dyspnea, cyanosis, and generalized edema. She had a single antenatal visit with no cardiovascular evaluation. Examination revealed finger clubbing, elevated jugular venous pressure, pitting edema, bibasilar lung crackles, and a systolic murmur. Vital signs were notable for heart rate 130 bpm, BP 110/70 mmHg, RR 25/min, and SpO₂ 80% on supplemental oxygen. Laboratory tests showed anemia and elevated CRP without fever. Imaging and echocardiography revealed a large ventricular and atrial septal defect with bidirectional shunt, severe tricuspid regurgitation, and estimated pulmonary artery pressure of 85 mmHg. DORV was suggested with the aorta arising from the right ventricle. A multidisciplinary team opted for vaginal delivery. Labor was induced with misoprostol (Bishop score 5) under continuous maternal monitoring. The patient delivered vaginally after 4 h. The newborn weighed 2.7 kg, with Apgar scores of 5 and 7 at 1 and 5 min, respectively. Postpartum, the mother received diuretics, blood transfusions, and antibiotics for elevated CRP. She stabilized by day seven and was transferred to cardiology for ongoing management.

**Discussion:**

This case highlights the challenges of managing pregnancy in women with ES and complex congenital heart defects like DORV. The successful vaginal delivery emphasizes the importance of a multidisciplinary approach, individualized treatment plans, and comprehensive monitoring to minimize risks. This case also demonstrates that vaginal delivery can be a viable option in selected patients when meticulously managed.

**Conclusion:**

Careful planning and multidisciplinary care can lead to favorable outcomes in pregnancies complicated by ES and DORV, despite their significant risks.

## 1. Background

Eisenmenger syndrome represents the advanced stage of long‐standing uncorrected congenital cardiac shunts, in which progressive pulmonary vascular remodeling leads to fixed pulmonary hypertension and reversal of intracardiac flow. Once established, the condition is associated with chronic systemic hypoxemia and limited ability to tolerate physiological stress.

Pregnancy introduces major hemodynamic changes, including increased blood volume, cardiac output, and oxygen demand, which are poorly tolerated in patients with Eisenmenger physiology and can precipitate rapid clinical deterioration. As a result, pregnancy in this group is associated with extremely high maternal and fetal risk and requires specialist multidisciplinary care [1].

Double outlet right ventricle (DORV) is a rare congenital cardiac malformation in which both the aorta and pulmonary artery arise predominantly from the right ventricle. This abnormal connection often coexists with septal defects and results in abnormal mixing of oxygenated and deoxygenated blood. When present alongside Eisenmenger physiology, it further worsens systemic oxygen delivery and increases the complexity of peripartum management [2].

Here, we report the successful management and delivery of a young female with Eisenmenger syndrome and DORV. This case highlights the critical role of a multidisciplinary team employing individualized monitoring and treatment strategies to achieve favorable maternal and neonatal outcomes [3].

## 2. Case Presentation

A 20‐year‐old woman, Gravida 1 Para 0, at 34 weeks of gestation, presented to the emergency department with progressive shortness of breath and generalized body swelling over several days. She had attended a single antenatal care visit at 12 weeks, during which no cardiovascular assessment was performed. She had no history of chronic illness, congenital heart disease, or prior surgeries, and denied alcohol or tobacco use. On presentation, the patient appeared cyanotic with finger clubbing and moderate respiratory distress.

Vital signs were heart rate 130 bpm, blood pressure 110/70 mmHg, respiratory rate 25/min, oxygen saturation 80% on supplemental oxygen, and temperature 37.2°C. Jugular venous pressure was elevated (~ 5 cm above the sternal angle). Peripheral pitting edema was present in both lower limbs, and lung auscultation revealed bibasilar crackles. Cardiovascular examination demonstrated a loud second heart sound with a systolic murmur at the left lower sternal border.

Laboratory investigations revealed anemia (Hb 8.2 g/dL, Hct 29.4%), leukocytosis (WBC 10.75 × 10^3^/mm^3^), and markedly elevated CRP (110 mg/L), though the patient was afebrile with no clear source of infection. Liver, renal, and thyroid function tests were normal, and serologies for HIV, Hepatitis B, and Hepatitis C were negative. Chest x‐ray showed cardiomegaly and pulmonary congestion (Figure 1), and ECG demonstrated sinus tachycardia with right ventricular strain pattern (Figure 2). Echocardiography revealed a large ventricular septal defect (VSD) and a large atrial septal defect (ASD) with bidirectional shunting, severe tricuspid regurgitation, and estimated systolic pulmonary artery pressure of 85 mmHg (Figures 3 and 4). Imaging to evaluate for DORV demonstrated the aorta arising predominantly from the right ventricle (Figure 5). However, full confirmation of DORV requires detailed visualization of the VSD‐to‐great vessel relationship. Pulmonary stenosis was not observed, and invasive assessment to determine reversibility of pulmonary hypertension was not performed, leaving the distinction between Eisenmenger syndrome and admixture physiology presumptive.

**Figure 1:**
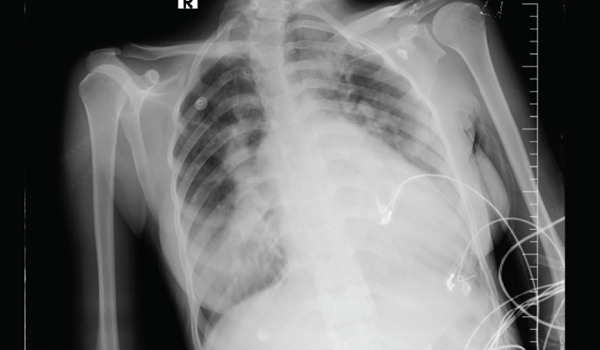
Chest x‐ray showing cardiomegaly with pulmonary vascular congestion, consistent with acute volume overload in the context of complex congenital heart disease and pregnancy.

**Figure 2:**
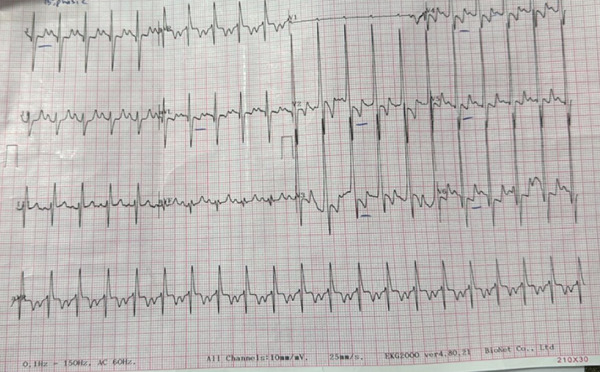
Electrocardiogram showing sinus tachycardia with features suggestive of right ventricular strain.

**Figure 3:**
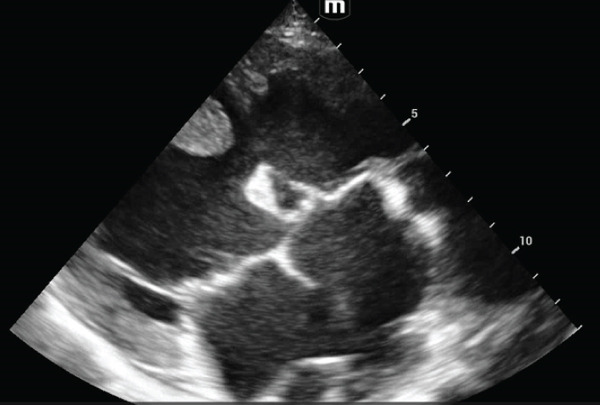
Transthoracic echocardiography, modified four‐chamber view, demonstrating a large ventricular septal defect and atrial septal defect. Cardiac chambers and septal defects are labelled in the figure.

**Figure 4:**
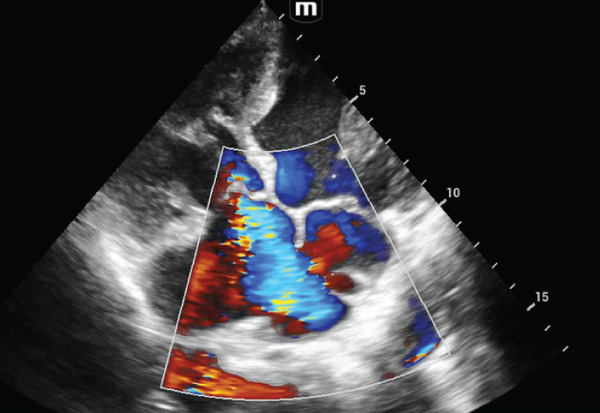
Transthoracic echocardiography with color Doppler showing intracardiac shunting through the atrial septal defect, with severe tricuspid regurgitation. The estimated systolic pulmonary artery pressure was approximately 85 mmHg.

**Figure 5:**
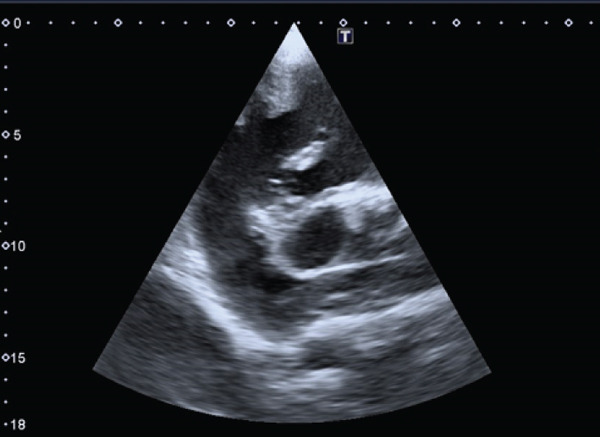
Echocardiographic view demonstrating the aorta arising predominantly from the right ventricle, supporting the diagnosis of double outlet right ventricle in association with ventricular septal defect.

The patient was managed by a multidisciplinary team including cardiology, obstetrics, anesthesiology, and neonatology. Given relative hemodynamic stability after initial stabilization, vaginal delivery was elected to avoid surgical/anesthesia‐related risks. Labor was induced with misoprostol, with a Bishop score of 5 prior to induction. Continuous monitoring included SpO₂, heart rate, and noninvasive blood pressure, with a target SpO₂ > 85%. Analgesia was carefully titrated to minimize cardiovascular stress.

The patient delivered vaginally 4 h after induction. The newborn weighed 2.7 kg, with an Apgar score of 5 at 1 min, improving to 7 at 5 min, and was admitted to the neonatal ICU for monitoring. Postpartum, the mother received diuretics for volume overload, blood transfusions for hemoglobin < 7 g/dL, and antibiotics in response to elevated CRP, despite no confirmed infectious focus. She was closely monitored in the ICU, and by Day 7 postpartum, she was hemodynamically stable and transferred to the cardiology inpatient service. Follow‐up included cardiology outpatient review within 1–2 weeks, ongoing pulmonary hypertension management, surveillance for Eisenmenger syndrome and DORV, and contraception counseling to prevent high‐risk future pregnancies.

## 3. Discussion

This case illustrates the complex interplay between Eisenmenger syndrome and DORV in the context of pregnancy, where both conditions contribute to severe limitations in cardiopulmonary reserve. The combination of fixed pulmonary hypertension and structural cardiac malformation creates a high‐risk hemodynamic state, particularly during labor and the immediate postpartum period.

Maternal mortality in Eisenmenger syndrome remains significantly elevated, largely due to risks of acute right ventricular failure, hypoxic crises, thromboembolic events, and arrhythmias [4]. The additional presence of complex congenital anatomy such as DORV further increases circulatory instability by promoting admixture physiology and worsening systemic desaturation [5].

Despite these challenges, the favorable maternal and fetal outcomes in this case make it a valuable contribution to the medical literature [6].

Although Caesarean section is frequently selected in such high‐risk pregnancies to avoid prolonged haemodynamic stress associated with labor, this approach is not without risk, particularly due to anesthetic complications and sudden changes in systemic vascular resistance. In carefully selected and clinically stable patients, vaginal delivery under strict monitoring may be considered within a tertiary care setting [7]. In the present case, the decision to proceed with controlled labor induction was based on multidisciplinary assessment and relative haemodynamic stability following initial optimisation. Short duration of labor, avoidance of prolonged second stage, and continuous maternal monitoring likely contributed to preventing acute decompensation. This approach highlights the importance of individualized delivery planning rather than a uniform mode‐of‐delivery strategy.

The postpartum period remains the most vulnerable phase in Eisenmenger syndrome due to abrupt autotransfusion from the contracted uterus and rapid shifts in preload and afterload. Careful intensive care monitoring during this period is essential to reduce the risk of late deterioration, which accounts for a significant proportion of maternal deaths [4].

Fetal outcomes in Eisenmenger syndrome are also poor due to chronic maternal hypoxaemia, with increased risks of intrauterine growth restriction, prematurity, and fetal loss [5]. In this case, continuous surveillance and supportive management contributed to a favorable neonatal outcome.

Overall, this case supports the possibility that, in highly selected patients with close multidisciplinary supervision, vaginal delivery may be achievable despite severe underlying cardiopulmonary disease. However, such decisions must remain highly individualized and restricted to centers with advanced maternal and critical care capability [7, 8].

## 4. Conclusion

Eisenmenger syndrome complicated by DORV in pregnancy represents an extremely high‐risk clinical scenario associated with significant maternal and fetal morbidity and mortality. This case demonstrates that, with careful multidisciplinary planning, close hemodynamic monitoring, and individualized decision‐making, successful vaginal delivery may be achievable in selected patients. However, such an approach should only be undertaken in specialized centers with appropriate obstetric, cardiology, and intensive care support, and Caesarean delivery should remain the preferred option in unstable cases.

## 5. Limitations

This case report has several limitations. First, the diagnosis of Eisenmenger syndrome was based on clinical presentation and echocardiographic findings, including severe pulmonary hypertension, bidirectional intracardiac shunting, and systemic hypoxaemia. However, right heart catheterisation was not performed; therefore, pulmonary vascular resistance and vasoreactivity could not be directly measured, limiting definitive haemodynamic classification.

Second, the diagnosis of DORV was based on echocardiographic features suggesting aortic origin from the right ventricle. Advanced imaging modalities such as cardiac MRI or CT angiography were not available, and therefore full anatomical characterization of the defect was not possible.

Third, detailed prepregnancy cardiac status and serial antenatal echocardiographic assessments were not available due to late presentation and limited antenatal follow‐up. Continuous invasive hemodynamic monitoring during labor was also not performed, and clinical decisions were guided by noninvasive monitoring and multidisciplinary judgment.

Finally, this is a single case report from a resource‐limited setting, and the favorable outcome cannot be generalized. Vaginal delivery in such high‐risk patients should only be considered after careful individual assessment in specialized centers with multidisciplinary expertise and intensive care support.

## Author Contributions

Osman Farah Dahir: conceptualization, investigation, data curation, writing—original draft, supervision, writing—review and editing. Said Abdirahman Ahmed: investigation, data curation, patient management, writing—review and editing. Ahmed Elmi Abdi: investigation, data curation, literature review, writing—review and editing. Ishak Ahmed Abdi: investigation, data curation, imaging interpretation, writing—review and editing. Ahmed Shafie Aden: methodology, validation, writing—review and editing. Mohamed Mukhtar Mohamed: clinical management (emergency care), investigation, writing—review and editing. Mohamud Mire Waberi: supervision, clinical oversight, writing—review and editing. Mohamed Omar Hassan: supervision, project administration, final approval of manuscript.

## Funding

No funding was received for this manuscript.

## Ethics Statement

Ethical approval was not required for this single anonymized case report in accordance with institutional policy. Written informed consent was obtained from the patient for publication of clinical details and accompanying images.

## Consent

Written informed consent was obtained from the patient for publication of this case report and associated clinical information.

## Conflicts of Interest

The authors declare no conflicts of interest.

## Data Availability

The data supporting the findings of this study are included within the article. Further information is available from the corresponding author upon reasonable request.
